# *Notes from the Field:* Large Measles Outbreak in Orthodox Jewish Communities — Jerusalem District, Israel, 2018–2019

**DOI:** 10.15585/mmwr.mm6918a3

**Published:** 2020-05-08

**Authors:** Chen Stein-Zamir, Nitza Abramson, Hanna Shoob

**Affiliations:** ^1^Jerusalem District Health Office, Ministry of Health, Israel; ^2^Hebrew University of Jerusalem, Faculty of Medicine, Hebrew University and Hadassah Braun School of Public and Community Medicine, Jerusalem, Israel.

During March 2018–May 2019, an outbreak of 4,115 measles cases occurred in Israel, following international importations, mainly from Ukraine. Approximately one half of the cases (2,202) occurred in residents of Jerusalem District, primarily in unvaccinated children in orthodox Jewish communities. The district’s population (1.25 million, approximately 14% of the national population) is 70% Jewish, approximately one third of whom are orthodox Jews. Children in those orthodox communities have lower rates of routine vaccination coverage; for measles vaccine, first dose coverge is 78.4%, compared with 90.1% among children in all other communities. Measles outbreak control in communities with long-standing inadequate vaccination coverage is challenging (*1*). Urgent response measures led to containment of this outbreak; however, sustaining vaccination coverage will require targeted interventions and resources.

The measles outbreak emerged in March 2018 in Israel’s Central and Northern districts. The first two cases in Jerusalem were in a student aged 20 years at a religious boarding school and a child aged 2 years. Both were unvaccinated and came to Jerusalem in August 2018 from measles-affected communities in the Northern district. Contacts included 300 of the student’s school contacts and 40 of the child’s relatives and neighbors. The outbreak quickly spread through the densely populated, low-income orthodox neighborhoods in Jerusalem District, where families have an average of seven children, and households might include 12–15 persons. Transmission intensified during the September–October Jewish high-holiday season, with 1,029 cases reported by October 31, 2018.

Overall, 2,202 cases were reported in Jerusalem District during August 2018–May 2019 (reported incidence = 176 per 100,000 population). Cases were confirmed by reverse transcription–polymerase chain reaction testing or detection of measles-specific immunoglobulin M in 708 (32%) patients and by epidemiologic linkage in 1,494 (68%). Approximately 8% of patients (176) were hospitalized (*2*). Two deaths occurred, one in an unvaccinated child aged 18 months, and the second in an immunocompromised adult, aged 82 years. Most cases (1,660, 75%) occurred in children aged <15 years. The highest reported incidence (1,174 per 100,000 population) occurred in infants aged <1 year, who accounted for 412 (19%) cases. Israel’s immunization schedule includes 2 measles-containing vaccine* doses at age 12 months and 6 years. Among 1,248 children with measles aged 1–14 years, 1,104 (88.4%) were unvaccinated; 128 (10.3%) and 16 (1.3%) had received 1 and 2 doses, respectively (Figure).

**FIGURE:**
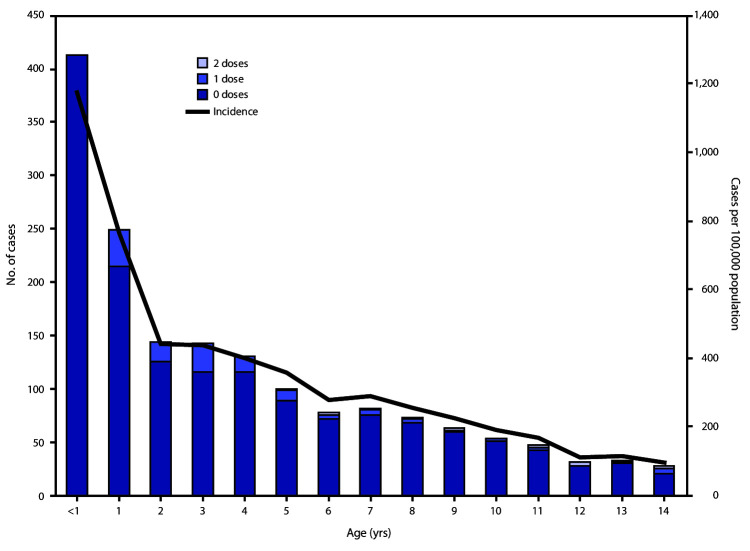
Age distribution of measles cases in persons aged <15 years (N = 1,660), by number of doses of measles vaccine received and age-specific measles incidence* — Jerusalem District, Israel, August 2018–May 5, 2019 * Cases per 100,000 population.

Jerusalem District Health Office teams conducted case finding and confirmation and contact tracing and distributed updates to health care providers. Measles patients and their parents were instructed to self-isolate; however, epidemiologic investigations revealed inadequate adherence. Infectious patients participated in crowded social events, attended child-care facilities, and used public transportation. Because of the large number of close contacts, tracing proved challenging. Outbreak response measures involved providing postexposure prophylaxis^†^ and conducting measles mass vaccination campaigns in the affected neighborhoods, targeting children and adolescents aged 1–14 years. The vaccination campaigns took place during September–December 2018 in outbreak neighborhoods in maternal-child preventive health services clinics (operated in 12-hour working shifts, in all clinics daily, exclusively for vaccinations), school health services, and a mobile vaccination unit. Culturally adapted approaches included dissemination of messages and outreach activities using telephone calls, community visits, and wall posters, and conveying information and guidance through word of mouth.

The emergence of a large number of measles cases and the very high incidence among young children in the orthodox communities engendered parental and societal anxiety and concern. Rabbinic leaders supported the vaccination campaign by issuing positive written statements, resulting in high levels of acceptance and compliance with control activities at the peak of the epidemic. Following the campaign, first-dose measles vaccination coverage in all maternal-child health clinics in orthodox neighborhoods increased from 76.3% in June 2018 to 96.1% in November. Since December 2018, the number of cases has decreased considerably. During October–December 2018, Jerusalem District accounted for 66% (1,652 of 2,486) of all measles cases in Israel; that percentage declined to 25% (248 of 969) during January–April 2019. As measles outbreaks continue to spread globally (*3*), achieving high, sustainable 2-dose coverage with measles-containing vaccine among age-eligible persons is essential to protect vulnerable groups, including infants too young for vaccination.

## Footnotes

* The vaccines used are measles-mumps-rubella-varicella for children aged 12 months–12 years and measles-mumps-rubella vaccine for all other age groups.

† Postexposure prophylaxis with measles vaccine was recommended for infants aged 6–8 months if it could be administered within 72 hours of exposure; otherwise, these infants were recommended to receive immune globulin until 6 days postexposure. Exposed persons aged ≥9 months were offered measles vaccine until 6 days following exposure. Immune globulin (within 6 days of exposure) was recommended for persons at high risk for measles complications, including infants aged <6 months, persons who were immunocompromised, and pregnant women.
